# Out of the Curricular Shadows: Revolutionizing Undergraduate Immunology Education

**DOI:** 10.3389/fimmu.2019.02446

**Published:** 2019-10-15

**Authors:** Heather A. Bruns, Jill Deaver, Louis B. Justement

**Affiliations:** ^1^Department of Microbiology, University of Alabama at Birmingham, Birmingham, AL, United States; ^2^Lister Hill Library of the Health Sciences, University of Alabama at Birmingham, Birmingham, AL, United States

**Keywords:** immunology, education, undergraduate, curriculum, major

## Abstract

Immunology has its developmental roots in understanding protection of the host from pathogens, leading to the development of vaccines and subsequently identification of soluble and cellular components of the immune system. Thus, immunology education has historically been tightly linked to infectious disease. Decades of research have demonstrated that the complexity and intricacies of the immune system are far greater than perhaps was once imagined. As a system that interfaces with all other organ systems in the body, it plays a key role in both maintaining health and causing life-threatening disease, thereby solidifying its importance in several clinical specialties beyond protective immunity. In the past decade, tremendous advances have taken place in which scientists and physicians have begun to harness the power of the immune system to create immunotherapies to fight cancer, inflammatory syndromes and autoimmune diseases. Thus, the argument can be made that training individuals in the field of immunology is becoming increasingly important. However, immunology is a highly conceptual discipline and understanding how the multiple cellular and soluble components of the immune system work in concert requires knowledge in a number of disciplines, including molecular biology, cell biology, genetics, and biochemistry. Time is needed for students to process, evaluate, and apply this information in meaningful ways. Concomitantly, knowledge in the field of immunology is expanding rapidly, bolstering the need for increased time in the curriculum to facilitate the ability of educators to convey information so that it can be effectively understood and applied. We propose that it is time for a renaissance in immunology education at the undergraduate level to better prepare individuals who will subsequently pursue careers in medicine, related health professions, and research. The purpose of this article is to discuss the current state of undergraduate immunology education with respect to its prevalence and how this compares to other biological disciplines, the need to develop robust immunology curricula at the undergraduate level and the importance of such programs in preparing students for pursuing postgraduate training in the health professions, and research-intensive careers.

## Introduction

The evolution of discovery in the field of immunology has been immense, highlighting the mechanistic intricacies of the immune system and opening opportunities to develop targeted therapeutic interventions based on understanding the fundamental molecular and cellular processes that regulate the function of the immune system. Education in the field of immunology has traditionally been restricted to graduate-level studies, but has not been widely adopted at the undergraduate level. An evaluation of undergraduate majors and programs that are focused on an immunology-intensive curriculum reveals that there are only a handful of such programs across the country, whereas there has been rapid growth of undergraduate programs in the biomedical field of neuroscience over the past 30 years. The comparison between immunology and neuroscience is particularly relevant because both systems are complex, interact with every other organ system in the body and play critical physiological roles in maintaining health, while at the same time having the potential to cause significant morbidity and mortality when they function abnormally. This raises the question of how and why neuroscience education has flourished at the undergraduate level, whereas immunology education has failed to gain traction. Although it might be logical to predict that this lack of immunology-focused programs may be compensated for by an increase in the number of undergraduate microbiology programs or an increase in the number of degrees conferred in this discipline, which is often associated with immunology, this is not the case. The total number of undergraduate microbiology programs and degrees conferred by such programs has remained relatively stable over the past 30 years.

The rapid expansion of discovery in the field of immunology has increased the demand for individuals who possess an in-depth understanding of the molecular and cellular processes that regulate the normal and pathophysiological function of the immune system, and who can effectively apply this knowledge to promote the health and well-being of individuals throughout the world. Concomitantly, there has been a call for evaluation and reform in the development of biology curricula that emphasizes multidisciplinary integration and the acquisition of competencies that extend beyond discipline-specific knowledge (1). Immunology, by nature, is interdisciplinary, requiring a knowledge of cellular and molecular biology, genetics, biochemistry, physiology, and anatomy. A strong case can be made that an undergraduate immunology major would exemplify the proposed reforms outlined in the Vision and Change report (1). Importantly, the shift to an emphasis on undergraduate training and the presence of robust undergraduate immunology programs could enhance the interest in, and number of students seeking immunology-related careers. We propose that the time has come for a reevaluation of the state of undergraduate immunology education. As a step forward, we have created an interdisciplinary major in immunology that provides both a broad-based curriculum, while at the same time providing a more in-depth focus on understanding the normal and pathophysiological function of the immune system. This major is designed to expose undergraduates to critical foundational and applied concepts in immunology at an earlier point in their educational experience and to prepare students for success at the next level, regardless of whether they choose to pursue a career in the health professions or in research.

### Why Immunology

Although it has been patently clear for a long time that the immune system plays a critical role in protecting individuals against infectious diseases based on centuries of research, it is now well-appreciated that the immune system can promote a wide range of debilitating diseases including autoimmunity and inflammatory syndromes that affect every organ in the body. Additionally, when misdirected, the immune system has the ability to cause significant suffering associated with allergies and asthma, which can often be life threatening. In the context of transplantation, the immune response to the transplanted organ presents a tremendous challenge from a clinical standpoint and negatively impacts the ability to provide safe and effective transplants. Thus, identifying ways to selectively block the immune response to a transplanted organ would represent a major advance in the field of transplantation that would dramatically impact the lives of over 100,000 individuals in the US who are waiting for a transplant at any given time (https://www.organdonor.gov/statistics-stories/statistics.html). With the increased use of immunosuppressive drug regimens associated with transplantation, autoimmunity, and cancer therapy, individuals suffer from increased risk of infection by opportunistic pathogens. This problem is accentuated by the fact that there is an ever-increasing risk from drug-resistant microbial pathogens and the emergence of new microbial pathogens in response to numerous technological, sociological and global factors. Finally, recent advances in the field of immunology clearly demonstrate the power of the immune system to fight cancer. Basic knowledge of how the immune system is regulated has led to the identification of checkpoint inhibitors that have been shown to be effective in terms of reactivating the immune system's ability to destroy cancer cells (2–4). Other approaches have taken advantage of the ability of immune cells to specifically identify tumor cells leading to their targeted destruction (5–7). It has only been within the last decade that researchers and clinicians have begun to understand how to harness the power of the immune system in the form of highly targeted and effective immunotherapeutic approaches designed to detect and destroy cancerous cells in the body. Thus, the immune system plays a critical role in both health and disease and there is a robust need for individuals who are focused on all aspects of the discovery and application pipeline from foundational studies to understand how the immune system works, to translation of foundational principles into novel immunotherapies and finally the application of immunotherapies in the clinical setting. Thus, one can make the argument that there is a significant need for individuals who understand how the immune system works and how that knowledge can be applied to improve health.

**Figure 1:**
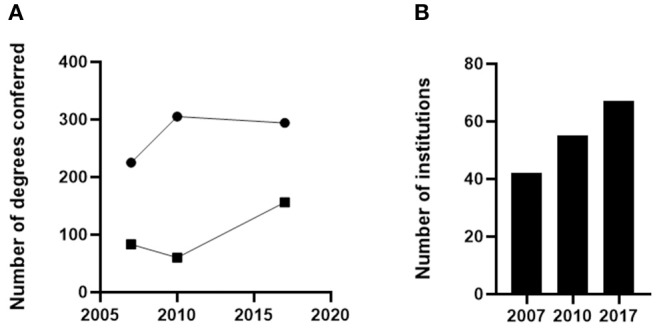
The number of doctoral (•) and master's-level (■) degrees conferred by U. S. institutions **(A)** and the number of U.S. institutions offering doctoral degrees **(B)** described as “Immunology” or “Microbiology and Immunology” or “Microbiological Sciences and Immunology” between 2007 and 2017.

Although one can make the case that knowledge of how the immune system functions has a high degree of relevance to careers in the health professions, biotech/pharma and research, it remains a challenging task to obtain employment data to categorically support the need to train more individuals in the field of immunology, primarily because the vast majority of careers that are most likely to benefit from knowledge of the immune system are not specifically defined by the terms “immunology” or “immunologist.” Nevertheless, one can obtain data on postgraduate education in immunology, as well as broad employment sectors in which immunology may be relevant to make the case that there is significant growth in those employment sectors that may be of interest to those individuals with training in immunology. With respect to postgraduate education, data from the National Center for Educational Statistics shows that between 2005 and 2017 there has been a modest increase in the number of MS and PhD degrees conferred by programs with an emphasis on Immunology (Figure 1A). In parallel, there has been growth in the number of postgraduate programs that focus on immunology during this same time period (Figure 1B). Thus, in contrast to the situation for undergraduate education in immunology, where there is little or no growth, data support the conclusion that postgraduate education in immunology is growing, albeit modestly.

Data from the U.S. Bureau of Labor Statistics also support the conclusion that employment sectors that could be of interest to those with knowledge of the immune are also growing at an above average rate over the next 10 years. The fastest growth sector from 2018 to 2028 is Healthcare and Social Services (8). This sector includes a wide range of occupations that would benefit from knowledge and training in immunology, so this is some indication that earning a degree in immunology may be of value in terms of obtaining a career in this sector. Additionally, there are a wide range of studies and reports from the American Association of Medical Colleges that clearly indicate that the US will face a shortage of approximately 122,000 physicians by the year 2032 (9). Once again, because the immune system plays a critical role in health and disease and has relevance to a wide range of clinical specialties, this suggests that education in immunology may be valuable for those who chose to pursue a career in the health professions. The U.S. Bureau of Labor Statistics predicts that employment in Health Diagnosing and Treating Practitioners that would include an emphasis on immunology will grow by 13% between 2018 and 2028, which is faster than the national average for all career categories (5%) (10). Growth in Medical Scientist careers will similarly increase by 8% between 2018 and 2028 (11). Data for the biotechnology sector from the IBISWorld Industry Report indicates that employment will increase approximately 4.6% between 2019 and 2024, which is slightly above the rate of growth for total employment in the US for that time period (12). Although these data do not specifically identify careers that rely on knowledge and skills in immunology, it can be argued that because of the important role that the immune system plays in health and disease and the increasing emphasis on harnessing the immune system for immunotherapies, that an education, which includes a focus on immunology may be beneficial to those interested in pursuing careers in the health professions, biotech, pharma, and research.

## Methods

### Use of College Navigator

Information on the number of institutions offering specific degrees and information on degrees conferred by major and institution in the 2017–2018 academic year was obtained through the National Center for Education Statistics (NCES; nces.ed.gov). College Navigator through NCES was used to identify numbers of institutions offering Bachelor of Science degrees in Neuroscience by searching “Neuroscience,” “Neurobiology and Neurosciences, Other;” in Microbiology by searching “Medical Microbiology and Bacteriology,” “Microbiology and Immunology,” “Microbiology general,” “Veterinary Microbiology and Immunobiology;” in Immunology by searching “Immunology,” “Microbiological Sciences and Immunology, Other,” “Microbiology and Immunology.”

### Use of IPEDS

An analysis of the number of neuroscience, microbiology, and immunology degrees conferred over time was performed using the Integrated Postsecondary Education Data System (IPEDS) through NCES. Provisional data for all U.S. institutions were searched for “total number” of “first major,” “Bachelor,” “Master's,” or “Doctor's” degree completions (award/degree conferred by CIP). The years 2017 and 2010 used the following degrees and CIP codes “Microbiology, General” and “Medical Microbiology and Bacteriology” (CIP codes 26.0502 and 26.0503) or “Immunology” and “Microbiology and Immunology” and “Microbiological Sciences and Immunology, Other” (CIP codes 26.0507, 26.0508, 26.0599) or “Neurobiology and Neurosciences” (CIP code 26.15). Prior degree categories and codes were slightly different from 2000 to 2009. For the 2007 data the following searches were done “Microbiology, General” and “Medical Microbiology and Bacteriology” (CIP codes 26.0502, 26.0503) or “Immunology” and “Microbiological Sciences and Immunology, other” (CIP codes 26.0507, 26.0599) or “Neuroscience” (CIP code 30.24).

### Literature Searches

Searches in the ERIC database were performed as follows for the indicated discipline.

#### Immunology, Results = 12

((Immunology AND (“Undergraduate Study” OR “Undergraduate Students”)) AND (“College Curriculum” OR “Curriculum Development” OR “Curriculum Design” OR “Curriculum Enrichment” OR “Curriculum Implementation” OR “Instruction” OR “Teaching Assignment^*^” OR “Teaching Experience^*^” OR “Language of Instruction” OR “College Instruction” OR “Instructional Development” OR “Courses” OR “Education” OR “Undergraduate Study” OR “Undergraduate Students” OR “major^*^” OR “program^*^”)).

#### Neuroscience, Results = 56

((Neurosciences AND (“Undergraduate Study” OR “Undergraduate Students”)) AND (“College Curriculum” OR “Curriculum Development” OR “Curriculum Design” OR “Curriculum Enrichment” OR “Curriculum Implementation” OR “Instruction” OR “Teaching Assignment^*^” OR “Teaching Experience^*^” OR “Language of Instruction” OR “College Instruction” OR “Instructional Development” OR “Courses” OR “Education” OR “Undergraduate Study” OR “Undergraduate Students” OR “major^*^” OR “program^*^”)).

#### Microbiology, Results = 98

((Microbiology AND (“Undergraduate Study” OR “Undergraduate Students”)) AND (“College Curriculum” OR “Curriculum Development” OR “Curriculum Design” OR “Curriculum Enrichment” OR “Curriculum Implementation” OR “Instruction” OR “Teaching Assignment^*^” OR “Teaching Experience^*^” OR “Language of Instruction” OR “College Instruction” OR “Instructional Development” OR “Courses” OR “Education” OR “Undergraduate Study” OR “Undergraduate Students” OR “major^*^” OR “program^*^”)).

Searches in the PubMed database were performed as follows for the indicated discipline.

#### Immunology; Results = 37

(((“Students”[Mesh] OR Undergraduate-student^*^[tiab]) AND Immunology[tiab])) AND ((“Curriculum”[Mesh] OR “Competency-Based Education”[Mesh] OR “Education”[Mesh] OR “Learning”[Mesh] OR “Teaching”[Mesh] OR Training-technique^*^[tiab] OR Pedagog^*^[tiab] OR Teaching-method^*^[tiab] OR Educational-technique^*^[tiab] OR Educational-activit^*^[tiab] OR Educational-method^*^ OR Short-term-courses[tiab] OR Training-program^*^[tiab] OR Academic-training[tiab] OR Workshop^*^[tiab] OR major^*^[tiab] OR program^*^[tiab])).

#### Neuroscience; Results = 130

(((“Students”[Mesh] OR Undergraduate-student^*^[tiab]) AND Neuroscience[tiab])) AND ((“Curriculum”[Mesh] OR “Competency-Based Education”[Mesh] OR “Education”[Mesh] OR “Learning”[Mesh] OR “Teaching”[Mesh] OR Training-technique^*^[tiab] OR Pedagog^*^[tiab] OR Teaching-method^*^[tiab] OR Educational-technique^*^[tiab] OR Educational-activit^*^[tiab] OR Educational-method^*^ OR Short-term-courses[tiab] OR Training-program^*^[tiab] OR Academic-training[tiab] OR Workshop^*^[tiab] OR major^*^[tiab] OR program^*^[tiab])).

#### Microbiology; Results = 103

(((“Students”[Mesh] OR Undergraduate-student^*^[tiab]) AND Microbiology^*^[tiab])) AND ((“Curriculum”[Mesh] OR “Competency-Based Education”[Mesh] OR “Education”[Mesh] OR “Learning”[Mesh] OR “Teaching”[Mesh] OR Training-technique^*^[tiab] OR Pedagog^*^[tiab] OR Teaching-method^*^[tiab] OR Educational-technique^*^[tiab] OR Educational-activit^*^[tiab] OR Educational-method^*^ OR Short-term-courses[tiab] OR Training-program^*^[tiab] OR Academic-training[tiab] OR Workshop^*^[tiab] OR major^*^[tiab] OR program^*^[tiab])).

Searches in the Scopus database were performed as follows for the indicated discipline.

#### Immunology; Results = 28

(“College Curriculum” OR Teaching OR Pedagogy OR major^*^ OR program^*^) AND (“Undergraduate Students”) AND Immunology.

#### Neurology; Results = 60

(“College Curriculum” OR Teaching OR Pedagogy OR major^*^ OR program^*^) AND (“Undergraduate Students”) AND Neuroscience.

#### Microbiology; Results = 55

(“College Curriculum” OR Teaching OR Pedagogy OR major^*^ OR program^*^) AND (“Undergraduate Students”) AND Microbiology.

## Results

### Immunology Left Behind

The career path decisions of students are influenced by a variety of factors (13). Chief among these factors are personal interest and academic ability, self-confidence (14) and importantly, for women or those from underrepresented groups, the availability of appropriate role models (15–17). Although conscious factors such as personal interest and academic ability contribute to student choices, learning experiences can subconsciously influence student perceptions of academic fields and subsequent career choices. Students who enter college with a strong sense of science identity, that is supported by a positive academic experience and perceived competence, tend to maintain a science identify and persist in a science career following graduation (18, 19). This raises the question of how and when to expose students to concepts in a given scientific field to enhance their positive perceptions of, and interest in that field. Traditionally, education in the field of immunology is not a major focus of science curricula at the high school or undergraduate college level. As a result, students are not exposed to the concepts in immunology in a meaningful way until they enter postgraduate educational programs.

In an effort to better appreciate the status of immunology education at the undergraduate level, we performed a number of comparisons to assess the number of undergraduate programs in immunology, the number of undergraduate degrees granted, and the extent to which immunology education is discussed in the literature. For these analyses, immunology was compared to two other disciplines; microbiology and neuroscience. Microbiology was chosen because historically, immunology has been associated with this discipline and in many instances, if immunology is taught, it is in the context of a microbiology undergraduate major, although curricula specific to immunology are often very limited and do not constitute a major emphasis in the vast majority of undergraduate microbiology majors. Secondly, neuroscience was chosen because from a biological and physiological perspective, neuroscience has a number of characteristics in common with immunology. The nervous system and the immune system are the only systems in the body that are comprised of a dispersed network of organs, tissues, cells, and soluble mediators that work in concert to regulate the function of that system. Moreover, both the nervous and immune systems interface with every other organ in the body, as well as each other. Thus, these systems are both highly complex entities that play a critical role in diverse aspects of health and disease. Both the nervous system and the immune system require students to understand conceptually how the system works as a whole, while at the same time appreciating the mechanistic interrelationships between the specific components that regulate the normal and pathophysiological function of the respective system. Both neuroscience and immunology have significant relevance in medicine and have the potential for significant technological and therapeutic breakthroughs that will impact the health of individuals throughout the world. For these reasons, the analyses conducted focused on comparing the status of undergraduate education in immunology to these two benchmark undergraduate majors.

**Figure 2:**
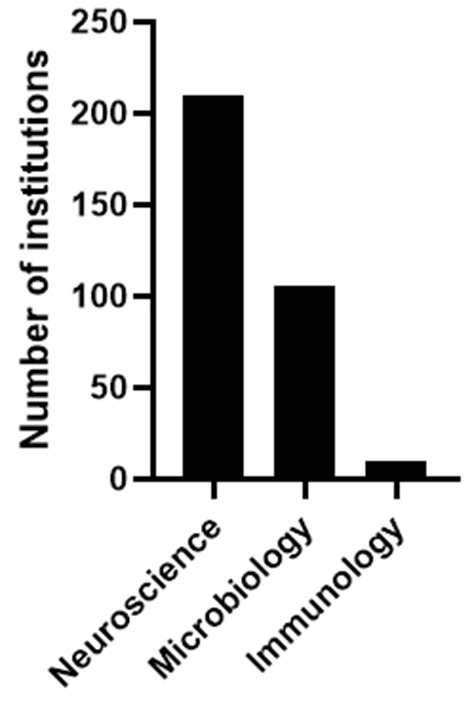
Comparison of the number of institutions offering Bachelor of Science degrees in the fields of Neuroscience (210), Microbiology (106), and Immunology (10) as of the 2017–2018 academic year. Data were collected from all U.S. institutions listed in the IPEDS data center.

Information on the number of institutions offering specific degrees was obtained through the National Center for Education Statistics (NCES; nces.ed.gov). It has been previously reported that undergraduate programs in neuroscience have grown rapidly over the past three decades (20). In 1986, only seven institutions reported having an undergraduate neuroscience program (major). This number more than tripled within a decade and tripled again by 2006 (25 institutions in 1996 and 90 institutions by 2006). Since 2006, the number of institutions offering undergraduate programs in neuroscience has continued to increase at a rapid pace, such that as of the 2017–2018 academic year, 210 institutions offered a Bachelor of Science degree in neuroscience (Figure 2). In contrast, there were 106 institutions that offered a Bachelor of Science degree in Microbiology, and only 10 institutions identified as offering an “immunology-related” major during the same academic year (Figure 2). The “immunology-related” majors were identified by including “Microbiological Sciences and Immunology, Other” and “Microbiology and Immunology.” Using only the search term “Immunology” yielded zero results. Furthermore, searching for a Bachelor of Science using “Immunology” as a keyword in internet-based college-finder software programs such as princetonreview.com and collegeboard.com yielded zero results.

**Figure 3:**
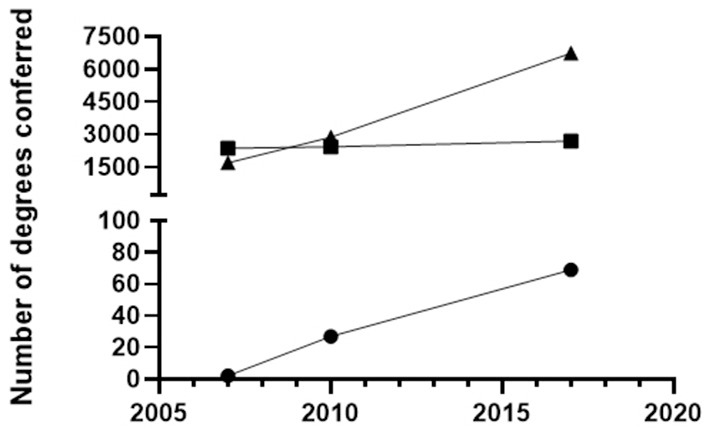
The number of Bachelor of Science Degrees in Immunology (•), Microbiology (■), and Neuroscience (▲) conferred by all U.S. Institutions listed in the IPEDS data center between 2007 and 2017.

An analysis of the number of neuroscience, microbiology, and immunology degrees conferred over time was performed using the Integrated Postsecondary Education Data System (IPEDS) through NCES. Similar to the number of neuroscience programs, the number of neuroscience degrees conferred since 2007 has rapidly increased (Figure 3). In comparison, the number of microbiology degrees conferred has remained relatively constant between 2000 and 2500 annually. The first immunology degrees conferred were in 2007. The number of immunology degrees conferred since then have been drastically fewer in number than the number of microbiology or neuroscience degrees.

**Figure 4:**
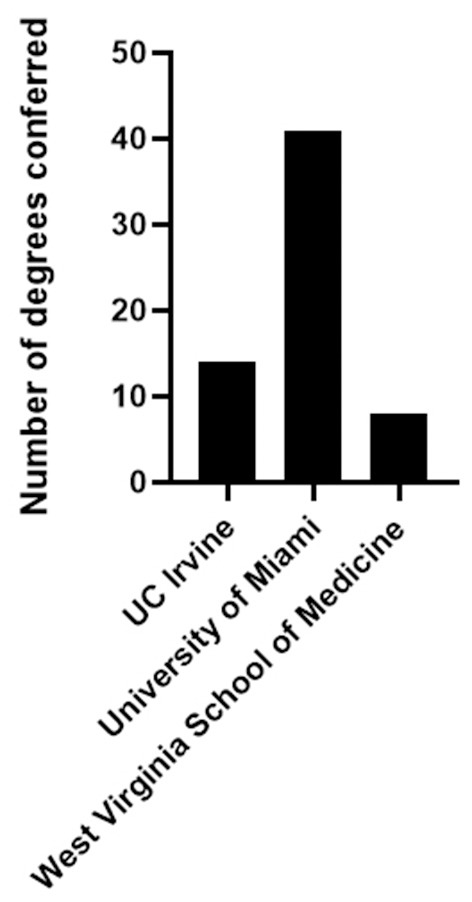
The number of Bachelor of Science Degrees in Microbiology and Immunology emphasizing immunology content conferred by U.S. accredited institutions in the 2017–2018 academic year.

Importantly, as mentioned above, a search for institutions with an “immunology major” yielded zero results. Thus, identifying “immunology-intensive” degree-granting programs, specifically, required further review of the Bachelor of Science course requirements for the 10 Microbiology and Immunology programs listed in College Navigator. This analysis revealed that the emphasis on immunology content was highly varied. Of the 10 programs identified by College Navigator in 2017, only three programs specifically offered a Bachelor of Science in Microbiology and Immunology that required three or more immunology courses to fulfill the degree requirements. In the 2017–2018 academic year, 69 degrees (B.S. in Microbiology and Immunology) between three institutions were conferred (Figure 4). All other programs listed were either majors in microbiology offering a maximum of two courses in immunology, concentrations available under other degrees that offered a maximum of two courses in immunology, or programs available only for graduate studies that were likely listed in error. It should be acknowledged that these numbers are approximate, as majors may exist that are not identified in IPEDS or College Navigator, such as the Immunology and Infectious Disease Major at Pennsylvania State University (https://vbs.psu.edu/majors/iid). Furthermore, we cannot account for programs that may offer concentrations in immunology that are optional and not required for the major. Regardless, the data obtained demonstrate that vast differences exist in the emphasis on undergraduate training in the fields of neuroscience, microbiology, and immunology.

In addition to the large offering of neuroscience and microbiology undergraduate programs, both the neuroscience and microbiology fields have education-focused journals (Journal of Undergraduate Neuroscience Education and the Journal of Microbiology and Biology Education, respectively) that support the development and dissemination of educational innovations. Furthermore, the Faculty for Undergraduate Neuroscience (FUN) held their first meeting in 1995 and created a set of guidelines for the development of undergraduate neuroscience programs, which are regularly reviewed and updated, and have served as a “blueprint” for the creation of new undergraduate majors by many institutions (21). Publications in neuroscience education have sought to identify the number and types of neuroscience programs and characteristics of the institutions offering them (20), how neuroscience is being taught at the undergraduate level (22, 23), and how best to assess undergraduate neuroscience programs (24, 25). Similarly, in an effort to support and initiate reform in undergraduate microbiology education, the American Society for Microbiology has created curriculum guidelines (https://www.asm.org/Guideline/ASM-Curriculum-Guidelines-for-Undergraduate-Microb) and also provides professional development resources (26). Similar efforts to promote undergraduate education in immunology at the national level are currently lacking, with perhaps the one exception being the American Association of Immunologists (AAI). AAI sponsors an Education Committee that broadly promotes immunology education, it hosts a special session at its annual meeting that focuses on immunology education, including curricular and pedagogical interventions in undergraduate, graduate, and medical education, the society has a resource page on its website (https://www.aai.org/Education/Teaching-Resources), and it has launched a new series “Teaching Tools” in its bi-monthly newsletter.

**Figure 5:**
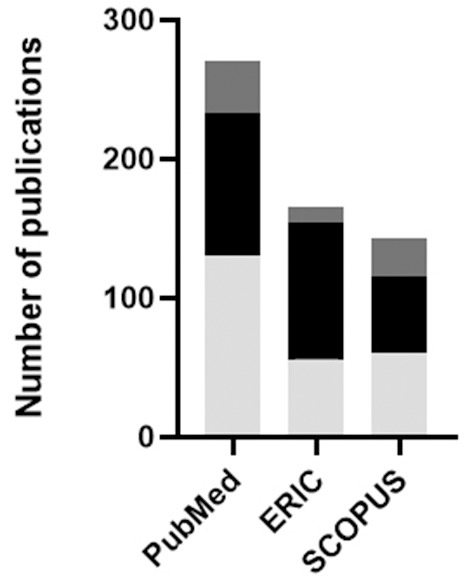
Education-related publications in the fields of immunology (dark gray), microbiology (black), and neuroscience (light gray) identified following searches of the PubMed, ERIC and Scopus databases. The search was performed on July 29th, 2019 and included all publications up to that date that met the search criteria.

Finally, a literature search was performed to map the current state of undergraduate education in the fields of immunology, neuroscience, and microbiology. The databases PubMed, Scopus, and ERIC were searched using the terms, “Immunology,” “Neuroscience,” and “Microbiology” as these fields relate to “Undergraduate Education” and “Curriculum” (Figure 5). Searches were broadened with appropriate synonyms specific to the individual databases, and reference lists for relevant articles were also searched. The purpose of this literature review was to determine the extent to which the field of undergraduate immunology education is represented in the literature and how this compares to microbiology and neuroscience. Although the results do not account for duplicate publications across databases, a total of 479 results were retrieved from the three databases. Approximately 20% of the citations retrieved focused on immunology, revealing a paucity of literature in this area compared to neuroscience and microbiology.

Based on these different analyses, it is readily apparent that undergraduate immunology education lags far behind neuroscience and microbiology. Microbiology programs have existed at the undergraduate level for decades and there has been only modest growth in the number of programs or in the number of degrees conferred during the past 30 years. In contrast, undergraduate neuroscience education has experienced robust growth in the same timeframe. At present, there are only a handful of programs that focus intensively on immunology education. It is possible that this is due to a lack of infrastructure to support such programs within the immunology community in the form of faculty groups and publications focused on undergraduate immunology education. It is possible that there are simply too few faculty who are trained to teach immunology and that undergraduate institutions do not have the necessary infrastructure to create new academic programs in this field. However, the fact that both microbiology and neuroscience are taught at a large number of undergraduate institutions, many of which are not directly affiliated with a school of medicine, tend to argue against this possibility. Finally, it is possible that the lack of growth in undergraduate immunology education may be due to a lack of awareness of the general public about immunology and its role in health and disease, which means that there is a lack of demand for such programs on the part of the “consumer” (i.e., the student). However, once again, one has to question how and why there appears to be a significant demand on the part of students to participate in microbiology and neuroscience programs, but not in the case of immunology.

## Discussion

### A New Path Forward

As demonstrated by the information discussed above, immunology, as a discipline, has made little headway in developing undergraduate programs and educational curricula as compared to its content counterpart, microbiology and the equally complex field of neuroscience. Student training in the field has been almost solely focused at the graduate and professional school level. We and others (27), propose a change from student training focused at the graduate and professional level to the development of educational curricula and training at the undergraduate level. As the pace of discovery in immunology accelerates and as novel immunotherapeutic interventions are developed, it can be argued that there is an increasing need for individuals who are trained in immunology and who will enter the workforce at multiple points in the pipeline, including those who are engaged in research-intensive careers, those who have the skills and knowledge to translate foundational discoveries into novel therapeutic interventions and finally, those individuals who are able to harness knowledge of the immune system and associated immunotherapies in the clinical setting (28). At most undergraduate institutions, courses in immunology are limited in number and scope. Traditionally, departments/programs in biology may offer one or two courses in immunology or host-pathogen interactions. Even at institutions with microbiology or microbiology and immunology departments/programs, immunology usually consists of one or two courses that are offered, with the exception of a few notable programs. The result is that the vast majority of students are exposed to immunology at most in one or two courses. Even then, the number of students entering graduate school or professional schools who have had an immunology course is in the 25–30% range. This raises several issues, including the fact that students are not likely to be able to fully appreciate how the overall immune system works from a conceptual perspective after a single overview or survey course, much less to understand how the system is regulated on a cellular and molecular basis, how the immune system is responsible for mediating serious disease processes, or finally, how it can be harnessed to fight disease. Because there is a clear lack of emphasis on immunology education at the undergraduate level, this significantly diminishes the number of individuals who are exposed to this discipline in an in-depth manner earlier in their educational experience and this may in turn impact the number of individuals who go on to pursue immunology-related careers.

At the University of Alabama at Birmingham (UAB), we have recently developed an undergraduate immunology major, the Undergraduate Immunology Program (UIP), which offers a Bachelor of Science degree, and is jointly sponsored by the Department of Microbiology in the School of Medicine and the Department of Biology in the College of Arts and Sciences. This major was purposefully developed not only to engage undergraduate students in the field of immunology but to promote the development of the core competencies outlined in The *Vision and Change in Undergraduate Biology Education* final report (1) and articulated by the National Postdoctoral Association (https://www.nationalpostdoc.org/page/CoreCompetencies). In addition to learning immunology content in the core courses of this major, students will develop transferable skills, including professionalism, communication, ethics, teamwork, and leadership. Through an intensive focus on undergraduate research, which is a requirement of the major, students will also develop critical thinking, problem solving, and analytical skills.

An outline of the 4 year curriculum is provided in Table 1. It is important to note that the UIP provides students with the opportunity to pursue a broad curriculum that includes the humanities and social sciences as well as core requirements in the sciences, including biology, chemistry, physics, and mathematics to meet the requirements for entry into professional schools. Course requirements for the major are listed in Table 2. In the first year of the major, students will focus on completing the university and science core requirements while taking an introductory seminar on current topics in immunology. Freshmen are introduced to several conceptual frameworks in this course that are carried on throughout their 4 year curriculum. These include the medically-relevant concepts of how immunology relates to vaccines, emerging infectious diseases, autoimmunity, allergy, transplantation, cancer, and immunotherapy. The goal is to provide context to help students understand how and why studying the immune system is relevant to health and disease.

**Table 1:** Undergraduate immunology program four-year curriculum.

**First term**	**Second term**
**Freshman**	
English	*Current Topics in Immunology (MIC 150)*
Math	Science core
Science core	Science core
University core	University core
Elective	Elective
**Sophomore**	
*Seminars in Immunology (MIC 250)*	*Introduction to the Immune System (MIC 275)*
Science core	Science core
Science core	Science core
University core	University core
Elective	
**Junior**	
*The Innate Immune System (MIC 401)*	*The Adaptive Immune System (MIC 402)*
*Research in Immunology (MIC 398)*	*Research in Immunology (MIC 398)*
Science core	Science core
Science core	Science core
University core	University core
**Senior**	
*Pathogen-Immune System Interactions (MIC 403)*	*Immune-mediate Diseases (MIC 404)*
*Research in Immunology (MIC 398)*	*Research Seminar in Immunology (MIC 492)*
Science core or Statistics	Science core
Science core	Science core
University core	

**Table 2:** Course requirements for the immunology major.

**Requirements**	**Hours**
**Biology**	
Introductory biology I	4
Introductory biology II	4
Genetics	3
Biology of microorganisms	4
**Chemistry**	
General chemistry I/general chemistry I laboratory	4
General chemistry II/general chemistry II laboratory	4
Organic chemistry I/organic chemistry I laboratory	4
Organic chemistry II/organic chemistry II laboratory	4
Fundamentals of biochemistry	3
**Physics**	
General physics I: mechanics	4
General physics II: electricity & magnetism	4
**Mathematics**	
Calculus I	4
Introduction to statistics or biostatistics	3
**Immunology**	
Current topics in immunology	1
Seminars in immunology	1
Introduction to the immune system	3
Foundations in immunology: the innate immune system	3
Foundations in immunology: the adaptive immune system	3
Foundations in immunology: microbial pathogen-immune system interaction	3
Foundations in immunology: immunologically-mediated diseases	3
**Undergraduate research (minimum of 6 h are required)**	
Undergraduate research in immunology & host defense	3
Undergraduate research seminar in immunology and host defense	3
Total hours	72

The second year allows students to continue working on core science courses and in the second semester students take the first course in the immunology core series, Introduction to the Immune System (MIC 275). This is an overview course designed to introduce students to basic concepts pertaining to the innate and adaptive arms of the immune response. This course provides the foundation upon which subsequent Foundations in Immunology 400-level courses, of which there are 5, will build. MIC 401-MIC 404 are required courses, whereas MIC 400, The Microbiome in Health and Disease, is optional.

In the third year, students take MIC 401, The Innate Immune System, followed by MIC 402, The Adaptive Immune System. These courses expand on the topics that were discussed in the Introduction to the Immune System course in order to develop the students' depth and breadth of content knowledge pertaining to the normal function of the immune system. Additionally, spanning the first three core courses (MIC 275, 401, and 402), is an embedded information literacy project. In MIC 275, students are introduced to databases, literature searches, and the concept of acquiring information from sources other than textbooks. Lectures and learning activities in MIC 401 require students to perform effective literature searches and foster the development of skills necessary for identifying and extracting relevant information and communicating information to peers. In MIC 402, students will be able to apply the skills they have developed through the completion of a presentation project. Throughout their sophomore and junior years, students in the UIP are expected to develop their written and oral communication skills through a series of individual and team-based activities. Examples of such activities include, writing articles to communicate to the lay public how immunology relates to disease (MIC 250), team-based presentations on techniques or therapies (e.g., vaccine development, flow cytometry, and monoclonal antibodies) that play a critical role in immunological research and treatment of diseases (MIC 275), team-based journal article presentations (MIC 401), and individual presentations covering “immunology in the news” topics (MIC 402).

In the fourth year, students will take the two remaining 400-level Foundations in Immunology courses; Microbial Pathogen-Immune System Interactions (MIC 403) and Immunologically-Mediated Diseases (MIC 404). In these courses, students will apply immunology concepts to understanding the interplay between microbial pathogens and the immune system as well as the importance of immune homeostasis in health and disease. These courses will reinforce the normal and pathophysiological principles pertaining to the immune system in a manner that is similar to what students might experience in medical school, thereby preparing them for the transition into that educational space. Going forward, it will be critical to incorporate additional content into the curriculum in MIC 404 that is focused on harnessing the immune system for the development of immunotherapies, as this is a rapidly growing area both in research and medicine. As mentioned above for the core courses taught in the sophomore and junior years, students will continue to be exposed to activities that reinforce their information literacy and communication skills. These educational themes will culminate in MIC 404, which is the Capstone course for the UIP, in which seniors will be expected to write a thesis and present their work orally.

Given the benefits students gain from research experience as an undergraduate (29, 30) and its influence on their attitude toward science and career choice (31), undergraduate research is a major component of the UIP. Students are expected to take a minimum of 6 credit hours of undergraduate research. In their sophomore year, students take the Seminars in Immunology course (MIC 250) in which they are introduced to research topics being pursued by faculty in the School of Medicine. This course is designed to reinforce many of the health-related themes that are built into the major and to encourage students to investigate research labs and meet with investigators in order to identify a lab with a suitable research project. Because this course is offered early in the sophomore year, we recognize that students may not have an in-depth appreciation of the immune system, much less concepts in research, including technical approaches. For this reason, we have modified the format of the Seminars in Immunology class recently to provide students with more opportunities to prepare for lectures from faculty. We have decreased the number of faculty lectures, and increased the number of lectures that talk about the specific areas of research that will be covered, as well as the techniques that are used. In this manner, students are given additional information to help them appreciate the stories that are presented to them by faculty. Additionally, we provide faculty with a rubric to ensure that they formulate a lecture that is primarily focused on the relevance of their research to health and disease, as opposed to being focused on technical details of the research itself.

Students in the UIP have the ability to choose from among over 100 faculty who are members of the Program in Immunology at UAB for research opportunities and are expected to join a laboratory no later than the beginning of their junior year. Starting in the fall of 2020, all freshmen in the UIP will be required to take a 1 credit hour course designed to introduce them to the principles of research. This course will be offered online and will cover the ethical conduct of research, safety training, the use of animals in research and human studies. Students will also learn what is expected of them in the research setting including, professionalism, record keeping, rigor and reproducibility, and other essential skills. Students are able to choose either undergraduate research or honors-level undergraduate research. In either case, students are encouraged to present their work at local, regional or national scientific meetings. For honors-level research, students are expected to write and defend a research thesis.

## Conclusion

Based on an analysis of the number of immunology programs/majors, the number of degrees conferred by such programs, the prevalence of articles in the literature that discuss curricular or pedagogical interventions in immunology, or the infrastructure available in the form of organized faculty groups, journals or other resources to support education in immunology, education in immunology does not appear to constitute a major focus at the undergraduate level. As a result, individuals are not readily able to gain an in-depth appreciation of principles in immunology or how those principles are applied to health and disease prior to entering graduate or professional school. This in turn may negatively impact the number of individuals who pursue immunology-related careers. This reality is in stark contrast to the state of undergraduate education in neuroscience, or microbiology. This realization begs the question; is it time for the immunology community to reevaluate the state of undergraduate education in immunology and to undertake a concerted effort to develop resources and programs to expose undergraduate students to this field more broadly? Hannum et al. have called for greater communication between undergraduate, graduate, and even professional level educators who teach immunology to start a dialogue regarding best practices for developing evidence-based learning outcomes to inform efforts to teach immunology at the undergraduate level. Moreover, it has been argued that immunology is truly an interdisciplinary field that inherently benefits from the cross fertilization of ideas and techniques from other areas in STEM (32, 33). A case in point is the fact that all sciences are now beginning to rely more heavily on informatics approaches, and immunology is no exception (33). This raises the potential for not only creating robust educational experiences in immunology, but interfacing those experiences with other STEM fields to create a truly interdisciplinary experience that prepares students to have greater flexibility to pursue a wide range of careers. The workforce demand for students with interdisciplinary degrees and the need for more individuals specifically trained in immunology (28) make a strong case for the immunology community to initiate a unified effort to develop robust undergraduate immunology programs. To date, only a handful of programs have been created that have an in-depth emphasis on immunology as a requirement. The UIP at UAB and other programs, including those at Penn State University, the University of Miami, West Virginia University, and the University of California, Irvine are such examples. These programs, in addition to the efforts of many other educators who oversee undergraduate courses in immunology offered through biology, microbiology or other science majors, provide a foundation upon which the immunology community can begin a serious dialogue to ask whether there is a need to drastically rethink how and when we should provide educational opportunities for students to expose them in an in-depth manner to the foundational and applied concepts of immunology. It is time to examine the need for a revolution in undergraduate education in immunology.

## Data Availability Statement

Publicly available datasets were analyzed in this study. This data can be found here: https://nces.ed.gov/.

## Author Contributions

Each individual named as an author made substantial contributions to the manuscript. LJ and HB wrote the manuscript and generated all figures. JD created and performed all literature searches. All authors approved the final version of the manuscript to be submitted.

### Conflict of Interest

The authors declare that the research was conducted in the absence of any commercial or financial relationships that could be construed as a potential conflict of interest.

## References

[1] AAAS Vision and Change in Undergraduate Biology Education: A Call to Action. Final Report. Washington, DC (2011).

[2] Alsaab HO Sau S Alzhrani R Tatiparti K Bhise K Kashaw SK. PD-1 and PD-L1 checkpoint signaling inhibition for cancer immunotherapy: mechanism, combinations, and clinical outcome. Front Pharmacol. (2017) 8:561. 10.3389/fphar.2017.00561 28878676 PMC5572324

[3] Philips GK Atkins M. Therapeutic uses of anti-PD-1 and anti-PD-L1 antibodies. Int Immunol. (2015) 27:39–46. 10.1093/intimm/dxu095 25323844

[4] Constantinidou A Alifieris C Trafalis DT. Targeting programmed cell death−1 (PD-1) and ligand (PD-L1): a new era in cancer active immunotherapy. Pharmacol Ther. (2019) 194:84–106. 10.1016/j.pharmthera.2018.09.008 30268773

[5] Jacoby E Shahani SA Shah NN. Updates on CAR T-cell therapy in B-cell malignancies. Immunol Rev. (2019) 290:39–59. 10.1111/imr.12774 31355492

[6] Wang H Kaur G Sankin AI Chen F Guan F Zang X. Immune checkpoint blockade and CAR-T cell therapy in hematologic malignancies. J Hematol Oncol. (2019) 12:59. 10.1186/s13045-019-0746-1 31186046 PMC6558778

[7] Minutolo NG Hollander EE Powell DJ Jr. The emergence of universal immune receptor T cell therapy for cancer. Front Oncol. (2019) 9:176. 10.3389/fonc.2019.00176 30984613 PMC6448045

[8] Bureau of Labor Statistics USDoL Employment by Major Industry Section. Washington, DC: USDoL (accessed September 17, 2019).

[9] Heiser S New Findings Confirm Predictions on Physician Shortage. Washington, DC: Association of American Medical Colleges (AAMC) (accessed September 17, 2019).

[10] Bureau of Labor Statistics USDoL Occupational Outlook Handbook, Physicians and Surgeons. Washington, DC: USDoL (accessed September 17, 2019).

[11] Bureau of Labor Statistics USDoL Occupational Outlook Handbook, Medical Scientists. Washington, DC: USDoL (accessed September 4, 2019).

[12] Curran J IBISWorld Industry Report NN001 Biotechnology in the US. IBISWorld (accessed September 25, 2019).

[13] Lent RW Brown SD Gail H Toward a unifying social cognitive theory of career and academic interest, choice, and performance. J Vocat Behav. (1994) 45:79–122. 10.1006/jvbe.1994.1027

[14] Schulz JF Thoni C. Overconfidence and career choice. PLoS ONE. (2016) 11:e0145126. 10.1371/journal.pone.0145126 26808273 PMC4726650

[15] Lawner EK Quinn DM Camacho G Johnson BT Pan-Weisz B Ingroup role models and underrepresented students' performance and interest in STEM: a meta-analysis of lab and field studies. Soc Psychol Educ. (2019) 22: 1–27. 10.1007/s11218-019-09518-1

[16] Gunter C. Science: it's a role model thing. Genome Biol. (2013) 14:105. 10.1186/gb-2013-14-2-105 23425527 PMC3663090

[17] Allen-Ramdial SA Campbell AG. Reimagining the pipeline: advancing STEM diversity, persistence, and success. Bioscience. (2014) 64:612–8. 10.1093/biosci/biu076 25561747 PMC4282132

[18] Eccles J Who am I and what am I going to do with my life? Personal and collective identities as motivators of action. Educ Psychol. (2009) 44:78–89. 10.1080/00461520902832368

[19] Robinson KA Perez T Nuttall AK Roseth CJ Linnenbrink-Garcia L. From science student to scientist: predictors and outcomes of heterogeneous science identity trajectories in college. Dev Psychol. (2018) 54:1977–92. 10.1037/dev0000567 30234346 PMC6152842

[20] Ramos RL Fokas GJ Bhambri A Smith PT Hallas BH Brumberg JC. Undergraduate neuroscience education in the U.S.: an analysis using data from the national center for education statistics. J Undergrad Neurosci Educ. (2011) 9:A66–70. 23493915 PMC3592722

[21] Wiertelak EP Hardwick J Kerchner M Parfitt K Ramirez JJ. The new blueprints: undergraduate neuroscience education in the twenty-first century. J Undergrad Neurosci Educ. (2018) 16:A244–51. 30254539 PMC6153019

[22] Hardwick JC Smith JS. Undergraduate neuroscience faculty: results from a survey of faculty for undergraduate neuroscience members. J Undergrad Neurosci Educ. (2010) 8:A101–7. 23493671 PMC3592718

[23] Pinard-Welyczko KM Garrison ACS Ramos RL Carter BS. Characterizing the undergraduate neuroscience major in the U.S.: an examination of course requirements and institution-program associations. J Undergrad Neurosci Educ. (2017) 16:A60–7. 29371843 PMC5777840

[24] Kerchner M Hardwick JC Thornton JE. Identifying and using ‘core competencies' to help design and assess undergraduate neuroscience curricula. J Undergrad Neurosci Educ. (2012) 11:A27–37. 23494749 PMC3592753

[25] Muir GM. Mission-driven, manageable and meaningful assessment of an undergraduate neuroscience program. J Undergrad Neurosci Educ. (2015) 13:A198–205. 26240530 PMC4521738

[26] Merkel SM. American Society for Microbiology resources in support of an evidence-based approach to teaching microbiology. FEMS Microbiol Lett. (2016) 363:fnw172. 10.1093/femsle/fnw172 27412169

[27] Hannum L Kurt RA Walser-Kuntz DR. Developing immunologists: a role for undergraduate education. Trends Immunol. (2016) 37:425–6. 10.1016/j.it.2016.03.008 27061265

[28] Bishop GA. Yes, we need PhD immunologists! Trends Immunol. (2015) 36:280–2. 10.1016/j.it.2015.03.003 25801018

[29] Seymour E Hunter A-B Laursen S DeAntoni T Establishing the benefits of research experiences for undergraduates in the sciences: first findings from a three-year study. Sci Educ. (2004) 88:493–594. 10.1002/sce.10131

[30] Lopatto D. Undergraduate research experiences support science career decisions and active learning. CBE Life Sci Educ. (2007) 6:297–306. 10.1187/cbe.07-06-0039 18056301 PMC2104507

[31] Harrison M Dunbar D Ratmansky L Boyd K Lopatto D. Classroom-based science research at the introductory level: changes in career choices and attitude. CBE Life Sci Educ. (2011) 10:279–86. 10.1187/cbe.10-12-0151 21885824 PMC3164567

[32] Stagaman K Martinez ES Guillemin K. Immigrants in immunology: the benefits of lax borders. Trends Immunol. (2015) 36:286–9. 10.1016/j.it.2015.03.008 25866281 PMC4420656

[33] Spreafico R Mitchell S Hoffmann A. Training the 21st century immunologist. Trends Immunol. (2015) 36:283–5. 10.1016/j.it.2015.04.001 25911462

